# Acute Gangrenous Cholecystitis During Pregnancy: A Case Report

**DOI:** 10.1155/cris/4562629

**Published:** 2026-10-21

**Authors:** Chansokha Soeur

**Affiliations:** ^1^ National Maternal and Child Health Centre Phnom Penh 120408 Cambodia

**Keywords:** acute cholecystitis, biliary disease, case report, gangrenous cholecystitis, open cholecystectomy, pregnancy

## Abstract

Acute cholecystitis is one of the most common nonobstetric surgical emergencies during pregnancy. Progression to gangrenous cholecystitis is rare but is associated with significant maternal and fetal morbidity. We report the case of a 26‐year‐old primigravida at 25 weeks’ gestation who presented with severe right upper quadrant pain, fever, and vomiting. Laboratory evaluation revealed elevated serum inflammatory markers. Transabdominal ultrasonography demonstrated gallbladder wall thickening, pericholecystic fluid, and biliary sludge. Despite 24 h of conservative management, her clinical condition deteriorated. Following multidisciplinary consultation, urgent open retrograde cholecystectomy was performed, revealing a gangrenous gallbladder. Postoperative recovery was uneventful, and she subsequently delivered a healthy term infant. Early recognition and timely surgical intervention are essential in the management of complicated biliary disease during pregnancy. This case highlights the importance of multidisciplinary care and supports current recommendations for operative management of severe or complicated acute cholecystitis during pregnancy.

## 1. Introduction

Biliary tract disease is a leading cause of nonobstetric abdominal pain during pregnancy, driven by hormonal and physiological changes that predispose to gallstone formation and biliary stasis [1]. Elevated progesterone levels reduce gallbladder motility and alter biliary flow, increasing the risk of cholelithiasis and acute cholecystitis [2]. Genetic and environmental factors may further contribute to gallstone formation in reproductive‐age women [3]. Although many cases are uncomplicated, pregnancy may increase the risk of recurrent biliary symptoms and progression to complicated disease when definitive treatment is delayed [1, 4].

Diagnosis may be challenging because nausea, vomiting, and abdominal discomfort can overlap with normal pregnancy‐related symptoms. Nevertheless, right upper quadrant pain, neutrophilia or a left shift, and characteristic ultrasonographic findings remain important diagnostic features of acute cholecystitis during pregnancy [5, 6]. Transabdominal ultrasonography is the preferred initial imaging modality because of its safety, accessibility, and diagnostic utility during pregnancy [7, 8].

Conservative management with analgesia, intravenous fluids, dietary modification, and antibiotics may initially be appropriate in selected patients; however, nonoperative treatment is associated with recurrent symptoms and may be followed by disease progression or readmission [9]. Delayed definitive treatment may increase the risk of complications, including gangrenous cholecystitis and perforation [10, 11]. Contemporary evidence generally supports surgical management when clinically indicated, with laparoscopic cholecystectomy considered the preferred approach for acute cholecystitis during pregnancy when technically feasible [12, 13].

Laparoscopic cholecystectomy can be performed during any trimester when indicated, and current surgical and obstetric guidance support timely operative management of symptomatic or complicated biliary disease during pregnancy [14–17]. Large cohort studies have demonstrated acceptable maternal and fetal outcomes following cholecystectomy during pregnancy [18]. Open cholecystectomy may remain appropriate in selected patients when laparoscopic surgery is not feasible or when severe inflammation, gangrenous disease, difficult anatomy, or other clinical circumstances necessitate an open approach [12, 15].

Gangrenous cholecystitis is an uncommon but potentially life‐threatening complication characterized by ischemia and necrosis of the gallbladder wall and may progress to perforation, peritonitis, or maternal sepsis. Prompt recognition, appropriate imaging, timely surgical intervention, and multidisciplinary management involving surgical, obstetric, and anesthetic teams are essential to optimize maternal and fetal outcomes [1, 3, 7].

We present a case of acute gangrenous cholecystitis at 25 weeks’ gestation that was successfully managed with urgent open retrograde cholecystectomy.

## 2. Case Presentation

A 26‐year‐old primigravida at 25 weeks of gestation presented to the emergency department with a 4‐day history of severe right upper quadrant abdominal pain, high‐grade fever (39°C), and persistent vomiting. She had no known medical comorbidities or previous surgical history.

On examination, she appeared acutely ill and tachycardic, with a pulse rate of 110 beats/min and a blood pressure of 90/60 mmHg. Abdominal examination revealed marked right upper quadrant tenderness with a positive Murphy’s sign. There was no pallor, jaundice, or peripheral stigmata of chronic liver disease.

Laboratory investigations revealed leukocytosis (WBC 20.3 × 10^9^/L) with neutrophilia (17.7 × 10^9^/L) and an elevated C‐reactive protein (CRP) level of 133 mg/L. Liver enzymes were mildly elevated (ALT 41 U/L and AST 39 U/L), whereas total bilirubin, amylase (25 U/L), and lipase (17 U/L) were within normal limits. The procalcitonin level was 0.971 ng/mL.

Transabdominal ultrasonography demonstrated gallbladder wall thickening (5 mm), pericholecystic fluid, and biliary sludge, findings consistent with acute cholecystitis. Obstetric ultrasonography confirmed a viable fetus with appropriate growth and normal fetal activity. The fetal heart rate was 150 beats/min before surgery, indicating reassuring fetal status.

The patient was initially admitted for conservative management with intravenous fluids, bowel rest, ceftriaxone and metronidazole, analgesia, and close maternal and fetal surveillance. Maternal monitoring included regular assessment of vital signs and clinical condition, while fetal well‐being was assessed by obstetric evaluation and fetal heart rate assessment as clinically indicated. Despite 24 h of conservative treatment, her abdominal pain worsened, and the systemic inflammatory response persisted. Given the lack of clinical improvement and concern for complicated acute cholecystitis, a multidisciplinary assessment was undertaken involving the general surgery, obstetric, and anesthetic teams. Urgent surgical intervention was recommended.

Considering the severity of the clinical presentation, suspected complicated cholecystitis, and localized peritonitis, an open cholecystectomy was selected. The decision to perform an open rather than laparoscopic approach was also influenced by the limited availability of an experienced laparoscopic surgeon at our institution. At 25 weeks of gestation, a midline laparotomy was chosen to provide adequate abdominal access and surgical exposure while allowing careful management of the enlarged gravid uterus and inflamed gallbladder.

The operation was performed under general anesthesia. Maternal hemodynamic status and oxygenation were monitored throughout the procedure by the anesthetic team. Fetal status was assessed before surgical intervention with a fetal heart rate of 155 beats/min. Intraoperative exploration revealed a markedly inflamed and gangrenous gallbladder, confirming complicated acute cholecystitis (Figure 1). A retrograde cholecystectomy was performed. Calot’s triangle was carefully dissected, and the cystic duct and cystic artery were identified, skeletonized, ligated, and divided. The dissection was then continued retrogradely along the gallbladder bed. The cystic duct stump was inspected, hemostasis was secured, and the liver bed was irrigated. A subhepatic drain was placed, and the abdomen was closed in layers. No intraoperative complications were encountered.

**Figure 1:**
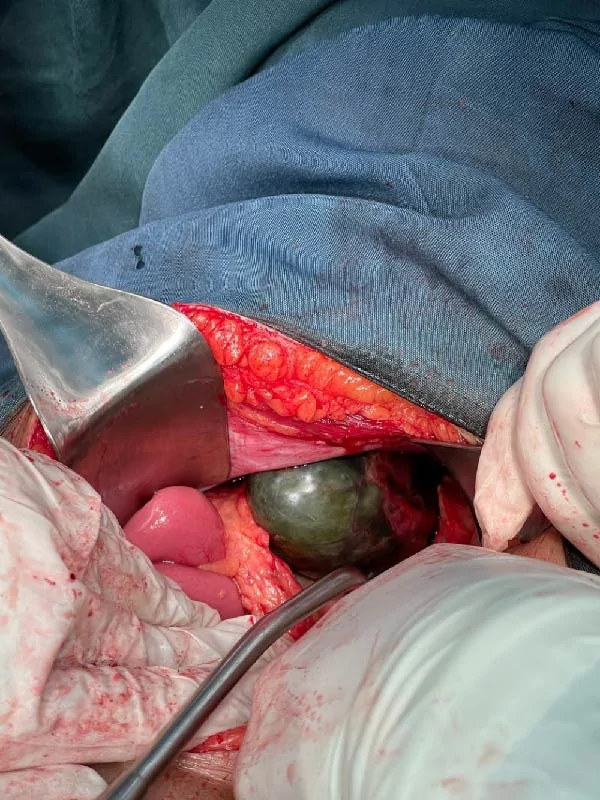
Intraoperative finding of acute gangrenous cholecystitis, showing a markedly inflamed gallbladder with extensive gangrenous and necrotic changes.

Following surgery, maternal clinical status and fetal well‐being were closely monitored during the postoperative period. Obstetric assessment, including the evaluation of fetal heart rate and fetal activity, was performed as clinically indicated. No evidence of fetal compromise or obstetric complication was reported during the postoperative hospitalization. By postoperative day 2, the inflammatory markers had improved substantially, with a WBC count of 7.4 × 10^9^/L and a CRP of 49.7 mg/L. The patient remained clinically stable and was discharged home on postoperative day 7.

Antenatal follow‐up was subsequently continued at her local health facility. Approximately 5 weeks after surgery, follow‐up obstetric ultrasonography demonstrated a gestational age of 30 + 2 weeks and an estimated fetal weight of 1569 g, with continued fetal viability and appropriate growth. The pregnancy subsequently progressed without any reported complications. At term, she delivered a healthy female infant weighing 3200 g by spontaneous vaginal delivery. Both the mother and newborn were well at follow‐up.

## 3. Discussion

Acute cholecystitis is one of the most common nonobstetric surgical conditions encountered during pregnancy. Its management requires careful consideration of maternal disease severity, gestational age, and fetal well‐being. Pregnancy‐related hormonal and physiological changes, particularly increased progesterone levels, reduce gallbladder motility and promote biliary stasis, thereby increasing the risk of gallstone formation and biliary disease [1, 2]. Genetic and environmental factors may further contribute to the development of gallstone disease in women of reproductive age [3].

The diagnosis of acute cholecystitis during pregnancy can be challenging because nausea, vomiting, and abdominal discomfort may overlap with common pregnancy‐related symptoms. Furthermore, physiological leukocytosis during pregnancy can reduce the specificity of the total white blood cell count as an inflammatory marker. Therefore, clinical assessment remains important, with particular attention to localized right upper quadrant pain and tenderness, fever, neutrophilia or a left shift, and characteristic imaging findings [5, 6]. Transabdominal ultrasonography is the preferred initial imaging modality because it is safe during pregnancy, widely available, and can demonstrate important features of acute cholecystitis, including gallbladder wall thickening, pericholecystic fluid, gallstones, and biliary sludge [7, 8].

Initial conservative treatment with intravenous fluids, analgesia, dietary restriction or bowel rest, and appropriate antibiotics may be considered in selected patients, particularly when symptoms are mild and there is no evidence of complications. However, conservative management does not address the underlying gallstone disease and may be associated with recurrent symptoms, readmission, or progression to complicated cholecystitis [8]. Persistent or worsening abdominal pain, ongoing systemic inflammation, recurrent attacks, obstruction, or evidence of severe gallbladder inflammation should prompt a reassessment and consideration of definitive surgical treatment. Previous reports have also described the failure of conservative management during pregnancy, including cases in which laparoscopic cholecystectomy was ultimately required [18]. These observations support close maternal and fetal monitoring and timely escalation of treatment when clinical improvement does not occur.

Current evidence supports the operative management of acute cholecystitis during pregnancy when clinically indicated. Laparoscopic cholecystectomy is generally considered safe during pregnancy and may be performed, when necessary, in any trimester, provided that appropriate surgical, obstetric, and anesthetic considerations are taken into account [13–16]. Comparative studies and systematic reviews have reported acceptable maternal and fetal outcomes following laparoscopic cholecystectomy, while nonoperative management may be associated with recurrent disease and additional hospitalizations [11, 12, 17]. Therefore, the choice between conservative and operative treatment should be individualized according to the severity of disease, response to initial treatment, gestational age, maternal and fetal condition, and the availability of appropriate surgical expertise and resources.

Gangrenous cholecystitis is a severe complication of acute cholecystitis characterized by ischemic necrosis of the gallbladder wall. It is associated with an increased risk of perforation, peritonitis, and systemic infection and generally requires prompt definitive management [7, 10]. In our patient, the initial presentation was concerning for severe acute cholecystitis, with high‐grade fever, hypotension, marked leukocytosis, neutrophilia, elevated CRP, persistent vomiting, and right upper quadrant pain. Despite 24 h of conservative treatment, her abdominal pain worsened, and the systemic inflammatory response persisted. Multidisciplinary reassessment by the general surgery, obstetric, and anesthetic teams led to the decision to proceed with urgent surgical intervention.

Intraoperative exploration demonstrated a markedly inflamed and gangrenous gallbladder, confirming the presence of complicated acute cholecystitis. Although laparoscopic cholecystectomy is generally preferred when feasible during pregnancy, an open approach may remain appropriate when laparoscopic expertise or resources are limited or when the clinical and operative circumstances favor an open procedure [13, 14]. In this case, the decision to perform open cholecystectomy was influenced by the severe gallbladder inflammation with localized peritonitis and the limited availability of an experienced laparoscopic surgeon at our institution. The midline laparotomy provided adequate access and exposure for definitive treatment in this particular clinical setting. Importantly, the decision was made following a multidisciplinary assessment, with consideration of both maternal and fetal safety.

The favorable postoperative course further supports the importance of timely definitive management in complicated disease. Following surgery, the patient showed clinical improvement and a substantial reduction in inflammatory markers, with the WBC count decreasing from 20.3 × 10^9^/L preoperatively to 7.4 × 10^9^/L by postoperative day 2. She was discharged in stable condition on postoperative day 7. Subsequent antenatal follow‐up demonstrated continued fetal viability and appropriate growth. The pregnancy progressed without reported complications, and she subsequently delivered a healthy female infant weighing 3200 g by spontaneous vaginal delivery at term. This favorable maternal and fetal outcome is notable given the severity of the initial presentation and the need for urgent abdominal surgery during the second trimester.

This case has several limitations. First, the original abdominal and obstetric ultrasonography images were unavailable; therefore, the ultrasound findings reported in this article are based on the documented written interpretations in the patient’s medical record. Second, formal histopathological confirmation of gangrenous cholecystitis was unavailable. The diagnosis was based on the clinical presentation, laboratory findings, documented imaging findings, and direct intraoperative observation of a gangrenous gallbladder. Third, as a single case report, this experience cannot establish the superiority of open over laparoscopic cholecystectomy or determine the optimal surgical approach for pregnant patients with complicated cholecystitis.

Overall, this case illustrates the potential for acute cholecystitis during pregnancy to progress to gangrenous disease despite initial conservative treatment. It emphasizes the importance of early recognition, close maternal and fetal monitoring, multidisciplinary decision‐making, and timely definitive treatment when clinical deterioration or complicated disease occurs. While laparoscopic cholecystectomy remains the preferred surgical approach when feasible, an open approach can be a reasonable alternative in selected clinical circumstances, particularly when severe inflammation is present or appropriate laparoscopic expertise and resources are unavailable. The successful continuation of pregnancy and term delivery of a healthy infant demonstrate that timely multidisciplinary management can result in favorable maternal and fetal outcomes even in severe biliary disease during pregnancy.

## Author Contributions

Chansokha Soeur conceived the study, managed the clinical case, collected and analyzed the data, performed the literature review, and drafted the manuscript.

## Funding

No funding was received for this study.

## Disclosure

The author has approved the final version for submission.

## Ethics Statement

The case report was reviewed by the local ethics and governance team at the National Maternal and Child Health Centre (NMCHC), Phnom Penh, Cambodia. Formal ethical approval was not required. Written informed consent was obtained from the patient for publication.

## Consent

Written informed consent for participation and publication of clinical details was obtained from the patient.

## Conflicts of Interest

The author declares no conflicts of interest.

## Data Availability

All data supporting the findings of this case report are included in the article. Additional anonymized information may be made available from the corresponding author upon reasonable request and with institutional approval.
